# Personal protective equipment and medical students in times of COVID-19: experiences and perspectives from the final clerkship year

**DOI:** 10.1186/s12909-023-04784-2

**Published:** 2023-10-26

**Authors:** Janina Straub, Anne Franz, Ylva Holzhausen, Marwa Schumann, Harm Peters

**Affiliations:** Dieter Scheffner Center for Medical Education und Educational Research, Dean´s Office for Study Affairs, Charité - Universitätsmedizin Berlin, Charitéplatz 1, 10117 Berlin, Germany

**Keywords:** Coping with uncertainty, COVID-19, Final-year clerkship, Impact on work readiness, Infection prevention, Medical student supervision, Personal protective equipment, SARS-CoV-2, Student safety, Training

## Abstract

**Background:**

The availability and correct use of personal protective equipment (PPE) to prevent and control infections plays a critical role in the safety of medical students in clinical placements. This study explored their experiences and perspectives in their final clerkship year with PPE during the COVID-19 pandemic.

**Methods:**

This qualitative study was based on social constructivism and was conducted in 2021 at the Charité - Universitätsmedizin Berlin. In three online focus group discussions, 15 medical students in their final clerkship year reported their experiences with PPE training and use during the COVID-19 pandemic. Data were recorded, transcribed and analysed based on Kuckartz’s approach to content analysis. We drew upon the a priori dimensions of the capability, opportunity, motivation - behaviour (COM-B) model as main categories as well as emergent issues raised by the study participants (subcategories).

**Results:**

In addition to the three main categories of the COM-B model, eleven subcategories were identified through inductive analysis. The study participants reported several factors that hindered the correct use of PPE. In the area of capabilities, these factors were related to learning experience with PPE in terms of both theoretical and practical learning together with later supervision in practice. In the area of opportunities, these factors included the limited availability of some PPE components, a lack of time for PPE instruction and supervision and inappropriate role modelling due to the inconsistent use of PPE by physicians and nursing staff. The area of motivation to use PPE was characterized by an ambivalent fear of infection by the SARS-CoV-2 virus and the prioritization of patient safety, i.e., the need to prevent the transmission of the virus to patients.

**Conclusions:**

Our study revealed several limitations pertaining to the enabling factors associated with the trainable behaviour “correct use of PPE”. The concept of shared responsibility for student safety was used to derive recommendations for future improvement specifically for the medical school as an organization, the teachers and supervisors, and students themselves. This study may guide and stimulate other medical schools and faculties to explore and analyse components of student safety in clinical settings in times of infectious pandemics.

**Supplementary Information:**

The online version contains supplementary material available at 10.1186/s12909-023-04784-2.

## Background

During the COVID-19 pandemic, the availability and appropriate use of personal protective equipment (PPE) by healthcare personnel has received global attention [1]. PPE plays an essential role in preventing COVID-19 and impeding the transmission of the virus [2]. Medical students, alongside healthcare workers, have contributed to the fight against the pandemic [3–5]; however, little attention has yet been given to their preparation for such tasks and the integration of such preparation into clinical settings. This limitation applies in particular to the experiences of medical students with educational instructions and supervision regarding the use of PPE in real-life clerkship placements during the COVID-19 pandemic. More information regarding this situation and the identification of potential gaps can guide us in the management of current and potential future pandemics. The purpose of this study is to explore medical students’ experiences with and perspectives on PPE during their final clerkship year during the COVID-19 pandemic.

The COVID-19 pandemic, which emerged in late 2019, escalated rapidly into a global health crisis, affecting communities around the world. With millions of confirmed cases and a staggering death toll [6], the pandemic was not only a threat to public health but also severely disrupted medical education [7]. After all, COVID-19 will not be the last pandemic to take its toll on our healthcare systems and the integration of medical students into the healthcare workforce during their clerkships. The World Health Organization estimates that between 80,000 and 180,000 healthcare workers worldwide had died from COVID-19 by May 2021 [8]. Frontline healthcare workers have been infected with the SARS-CoV-2 virus more frequently than members of other occupational groups, especially during the early waves of the pandemic [9, 10]. Medical students, who represent a relevant part of the healthcare workforce during the COVID-19 pandemic, have exhibited overall high seroprevalences of SARS-CoV-2 compared with other groups of healthcare workers and the general population [11, 12]. This situation may raise questions regarding appropriate measures to ensure the safety of students in their clinical placements in light of the associated infectious hazards.

Student safety is a key objective in clinical placements that must be balanced with the needs and benefits of students’ learning in a real working environment [13]. Although medical students have previously been trained in infectious and hazardous environments, COVID-19 added a new dimension to their active involvement in practical hospital training [14]. Particularly during the early stages of the COVID-19 pandemic, there was an urgent need to reorient the practical training of medical students in light of the many uncertainties regarding the routes of viral transmission or the unknown long-term risks following infection. Part of this equation is the fact that medical students in their clerkships should be viewed as relatively early learners with limited clinical knowledge, skills and experience. Active educational support, supervision and feedback are paramount for medical students in clinical placements, as they have not yet become qualified health professionals [15]. Responsibility for student safety in potentially hazardous clinical environments is a multidimensional construct and should be viewed as a shared responsibility among medical schools and their affiliated teaching institutions, the teaching and supervising faculty, and medical students themselves [16]. As part of their acute responses to the COVID-19 pandemic, many medical schools removed students from patient care and made student safety their top priority [17, 18]. As the pandemic progressed, clinical placements continued, balancing the increased risk of infection with enhanced safety measures [19]. In this context, innovative teaching methods, such as simulation-based exercises, gained new importance, especially for the practical training of medical students [20].

As the pandemic unfolded with unprecedented speed, the availability and the appropriate use of PPE became a critical factor in the prevention of SARS-CoV-2 infections in the clinical environment [21], in addition to basic hygiene measures and patient testing and isolation. The correct use of PPE together with consistent hygiene measures has been shown to minimize the risk of SARS-CoV-2 infection [22]. As vaccination was not available until the pandemic was well underway, the ready availability and appropriate use of PPE played a key role in adjusting medical education to the needs of the pandemic. The appropriate use of PPE in clinical practice is a trainable albeit complex skill; the relevant training includes when and where to use such equipment, the correct choice of equipment, and the correct processes for donning and doffing PPE [23]. To date, only scant information regarding the use of PPE and the preparation of medical students in clinical placements in times of COVID-19 has been reported. One study conducted in the United Kingdom reported that more than half of the participating medical students had not received sufficient training in infection control and had therefore experienced higher levels of uncertainty [24]. Therefore, there is a strong need for more information regarding PPE instruction and skills training for students prior to entering placements that feature potential exposure to SARS-CoV-2 as well as for support, guidance, supervision and feedback during such placements. The identification of potential gaps in this context can guide improvements in student safety during the current and potential future pandemics.

The aim of this study is to explore the experiences and perspectives of medical students in their final clerkship year regarding PPE during COVID-19 using a qualitative focus group approach. In this study, we conceptualize the appropriate use of PPE as a trainable behaviour according to the capability, opportunity, motivation - behaviour (COM-B) model, which serves as the basis for the behaviour change wheel [25]. Accordingly, we analyse medical students’ experiences with practising PPE in relation to enabling factors in terms of the dimensions of capability, opportunity and motivation.

## Methods

### Setting

This study was conducted in 2021 at the Charité - Universitätsmedizin Berlin (Charité), Germany. For all medical students in Germany, the final year of training follows five years of study. The final year is divided into three tertials, i.e., one in internal medicine, one in surgery and one elective of the students’ choice [26]. The overall aim of the final clerkship year is to involve medical students actively in patient care with increasing levels of autonomy with the aim of preparing them for the residency that follows graduation [27]. The Charité is one of the largest teaching hospitals in Germany; in this context, the final clerkship year can be completed either at the Charité campuses or at one of its affiliated regional teaching hospitals. Final year clerkship students at the Charité can come from its own undergraduate programme or from other German medical faculties.

Since the beginning of the COVID-19 pandemic in Germany in January 2020, the Charité has played a key role in the wide-ranging efforts made to contain and manage the pandemic in this country; for instance, it has been a leading institution in terms of patient care, the development of guidelines, research and advising the government [28]. In March 2020, the German government and the federal states, like many other countries worldwide, decided to impose far-reaching restrictions on public life to contain the pandemic [29]. For German medical faculties, these restrictions required face-to-face courses to be converted to digital formats within a very short time to deliver undergraduate medical programs. Simultaneously, it was decided that final-year clinical placements should continue. At the Charité, hospitals decided to separate COVID-19 patients from noninfected patients to treat them separately. Accordingly, final-year medical students were not only involved in the clinical care of non-COVID-19 patients but also worked in COVID-19 wards and emergency departments, including intensive care units. Regular COVID-19 screening of staff at multiple testing sites to which students had access was established early in this process.

Prior to the start of their final clerkship year, medical students were provided with an interactive educational video via the Charité e-learning platform that addressed general characteristics of infection prevention and control and provided basic information regarding SARS-CoV-2 and an overview of the correct use of PPE. Students were required first to watch the video and then to take a test; the estimated time to complete both was approximately 90 min. The online certificate provided upon successful completion of the test was required to be presented in the ward on the first day of their final clerkship year. Students were also expected to familiarize themselves with the hygiene guidelines available on the Charité intranet, which included a more detailed video regarding the process of putting on and taking off PPE.

### Study design

This study is underpinned by social constructivism, being the epistemology of how knowledge is acquired and justified by people constructing and reflecting their own understanding of the world [30]. In this study, final-year medical students can be viewed as active participants who jointly construct experiences and meanings with respect to the use of PPE as a trainable behaviour [31, 32]. As focus groups are aligned with the constructivist paradigm and are effective with respect to exploratory data collection, they represent the data collection method of choice that allows this study to explore medical students’ experiences with and behaviours and perspectives regarding the use of PPE, a topic that would benefit from exploration based on the synergistic and dynamic focus group format [33–35]. The focus groups were conducted online as a result of general prevention measures related to the ongoing COVID-19 pandemic [36]. The study, including the data protection policy, was approved by the responsible ethics committee of the Charité (application number EA4/149/21). An additional data protection vote was not considered to be necessary. We followed the Consolidated Criteria for Reporting Qualitative (COREQ) Research [37] in reporting the characteristics of this qualitative study.

### Study participants

Purposeful maximum variation sampling was employed for this study, and the principle of data saturation during an iterative process was embraced to balance the breadth and depth of the research topic [38]. Inclusion criteria were medical students who had completed at least one tertial during their final clerkship year at the Charité or at one of its teaching hospitals. The final tertial should not have been completed longer than 12 months ago, thus allowing for the inclusion of medical students from the early period of the pandemic in 2020. Study participants were recruited via the e-mail list of all final-year medical students and through calls made on social media channels. Participation was voluntary and no compensation was provided.

Prior to the focus group discussions, the study information and data protection policy were sent to the study participants alongside a short questionnaire used to collect sociodemographic data. Participants were required to provide written consent to participate in a focus group discussion.

### Focus group discussions

A discussion guide was developed in advance using a systematic and step-by-step approach. The author JS produced a draft of the discussion guide that mapped the main themes in the context of this study. This guide consists of open-ended introductory questions, which are supplemented by more specific follow-up questions in each thematic area. The draft was then iteratively discussed within the author team and in a methodological colloquium with educational research experts at the Charité with the aim of improving the relevance and structure of the questions. After incorporating the resulting feedback, the discussion guide was pilot-tested for comprehension and clarity by a medical student representing the target group for the later focus group discussions. This resulted in further minor adjustments to some of the wording. The medical student did not participate in the study focus groups. The final discussion guide can be found in appendix 1.

The online focus groups were conducted using MS TEAMS  (Microsoft Deutschland GmbH, Munich, Germany). The author JS moderated the group discussions; the moderator had no direct acquaintance relationships with the study participants or any relationship to their subsequent professional lives. Each focus group began with a round of introductions so that participants could get to know each other. Another intention was to create an open and safe social environment in the virtual space so that the participants could feel a sense of belonging and cohesion, and feel comfortable sharing information and expressing their opinions freely in the discussion [39]. The substantive discussion was recorded using external audio recording equipment; these audio recordings were subsequently transcribed verbatim by the author JS.

**Fig. 1:**
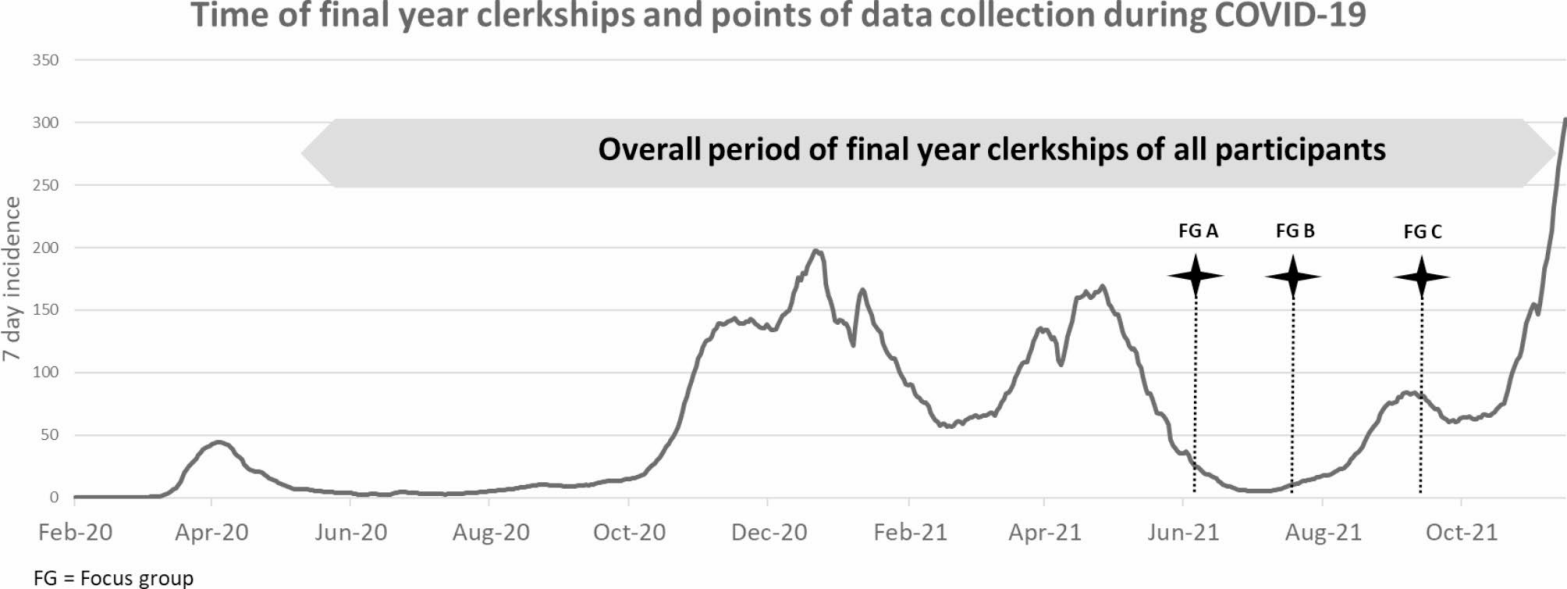
(own illustration): Seven-day incidence of SARS-CoV-2 in Germany during 2020/21 including the overall period of final-year clerkships of all study participants (grey) and points of data collection in focus groups (FG = focus group) | Source of 7-day incidence: NPGEO Corona based on the Robert Koch Institute

Figure 1 illustrates the overall timing of the final-year placements of all study participants and the dates of the focus groups over the course of the COVID-19 pandemic.

### Data analysis

The analysis followed Kuckartz’s suggestions for qualitative content analysis using a deductive approach [40, 41]. First, relevant sections of the transcribed audio recordings were analysed using a deductive procedure. The COM-B model, which was used as a framework for the deductive approach, includes three components: (1) *capability*, (2) *opportunity,* and (3) *motivation* [25]. These three components served as the main categories for the analysis. The COM-B model is viewed as a prerequisite for understanding behaviour and serves as the basis for the behaviour change wheel. This approach has previously been shown to be useful in the analysis of qualitative data and facilitates a structured analysis of the factors that determine the experiences and behaviour of young medical professionals with regard to the use of PPE and the identification of actions that could lead to a change in behaviour [42].

Quotations from participants regarding their experiences of dealing with PPE during their final-year placements were assigned to the main categories derived from the COM-B model and grouped accordingly. Subcategories were then developed inductively within the main categories based on this material. The author JS created a preliminary category system with a set of coding guidelines that was then critically reviewed by the researcher AF, who has extensive experience in qualitative research. The feedback was discussed and incorporated in the following step. During the analysis of three focus group discussions, data saturation was achieved. All transcribed data were then coded based on the final category system; consensus was reached in the case of differing assignments of codes. Finally, overlapping categories or subcategories were regrouped or redefined.

The original quotations presented in this article have been translated into English by the authors. All quotations have been anonymized. Quotations are marked with letters (A-C) to indicate the focus groups, and additional numbers (1–5) have been added to distinguish among the different participants within each focus group. As part of a member check, the findings of the study were shared with the focus group participants by e-mail, and their feedback was taken into account.

## Results

Three online focus group discussions including a total of 15 participants were held between June and September 2021. Table 1 shows the sociodemographic data of the study participants. The study participants participated in one of the three focus group discussions (n = 5 in each group). The focus group discussions lasted 72, 78 and 85 min.

**Table 1:** Sociodemographic data of study participants

		n = 15
**Age** (mean, range)	Years	27 (24–32)
**Gender**	Female n (%) Male n (%) Not specified n (%)	11 (73) 3 (20) 1 (7)
**Home medical school**	Charité students n (%) External students n (%)	13 (87) 2 (13)
**Tertials completed** (multiple answers possible)	Internal medicine n (%) Surgery n (%) Elective n (%)	10 (67) 5 (33) 12 (85)

Eight students (53%) participated in these focus groups during their final-year clerkships at Charité or a teaching hospital associated with Charité, whereas seven study participants had already finished medical school at the time of the study.

As the main categories were derived from the literature, it was necessary to operationalize them in accordance with the topic of this study. In addition to the three main categories of the COM-B model, a total of eleven subcategories emerged inductively from the dataset (see Table 2). Hereafter, our main findings are reported in a summary description of the discussed contents with supporting quotations.

**Table 2:** Overview of the category system with the main categories derived from the COM-B model and the assigned subcategories

COM-B - Categories		Subcategories (developed based on study material)
**Capability**		• Theoretical knowledge of PPE • Initial practical instruction regarding PPE in the hospital • Supervisors for the application of PPE in clinical routine • Suggestions for improvement
**Opportunity**		
	**Physical opportunity**	• Availability of PPE
	**Social opportunity**	• Inconsistent use of PPE • Communication and information flow • Time required for PPE instruction and supervision • Influence of the course of the pandemic on the handling of PPE
**Motivation**		• Fear and uncertainty • Prioritization of patient safety

### Capability

This category explores the knowledge, skills and abilities required to engage in the behaviour associated with the appropriate use of PPE. Four subcategories were identified.

Regarding the **theoretical knowledge of PPE**, the study participants mentioned the instructional video provided by Charité prior to starting their final clerkship year as the main source. However, they had mixed views regarding this video: while some participants claimed that the video offered them time for reflection at home, others did not find it to be helpful.

*C2: Well, I watched also these hygiene instructions from the Charité, but nobody remembers them, they are much too long. It somehow goes on for an hour and a half, where you think to yourself: “What should I take away from it?” (FG C, 24)*.

Participants further reported that they had also used digital learning opportunities not provided by the university, such as YouTube or learning platforms, and had found them to be helpful.

With regard to the **initial practical instruction regarding PPE in the hospital**, the study participants’ experiences ranged from no practical instruction in the wards at all to detailed and structured instructions. Participants agreed that the latter instruction took place in the intensive care units for COVID-19 patients in particular.

*A2: So, for that reason alone, the resident physician definitely went along the whole way with us two or three times and accompanied us completely and step by step while undressing [i.e., removing PPE]. But that ward was nearly completely specialized for COVID. We then had instructions on how to put on [PPE] adequately; in addition, they had proper procedures there. (FG A, 20)*

In contrast, the majority of participants reported that they had not received any practical instruction regarding PPE in normal wards. In this context, practical knowledge on PPE was mostly presumed, and there was no inquiry into (non-) existing competences in these wards.

*A5: In the ward itself, I didn’t get any special instruction; it [the PPE] was just placed in front of the patients’ rooms. It was rather assumed that one knew how to put on the protective clothing properly. (FG A, 33)*

Different professional groups were named as **supervisors for the application of PPE in routine clinical practice**. It is worth mentioning that nurses were mentioned more frequently than medical colleagues.

*C5: I think that it is somehow not quite clear who is responsible, so in my case, it was as well the case that often the nursing staff pointed something out to me, although it should actually have come from the physician´s side also. (FG C, 28)*

Other professional groups, such as hygiene officers or colleagues from the department of microbiology, were also occasionally mentioned as persons who were in charge of supervision regarding the correct use of PPE.

For ease of understanding, the subcategory “suggestions for improvement” is presented at the end of this section.

### Opportunity

This category explores the external factors that make execution of the behaviour in question possible—in our case, the physical and social opportunities that make the appropriate use of PPE possible. Five subcategories were identified.

The (physical) **availability of PPE** was mentioned primarily by participants who had first-hand experience of material shortages at the start of the pandemic. In particular, these participants addressed the strict rationing of face masks and the need to address frequent changes in PPE models. In particular, it was mentioned that both students themselves and staff in the clinical setting were required to improvise when face masks did not fit tightly. Thus, “different styles” were used to ensure that the face mask fits adequately.

*A2: […] we had new masks every week, so we had quite different models, up to these construction workers’ protective masks, and they were simply too big for my face, and then I taped them myself. So, with [medical] adhesive tape, no sense at all of whether that improved it. But I felt a bit safer when I went to the first COVID patients, in any case […] (FG A, 40).*

Regarding social opportunities, four subcategories were identified, which pertained to the sociocultural environment in the hospital as well as factors over which students have little to no control. The **inconsistent use of PPE** was a major topic of discussion. Inconsistency in this context mainly refers to the variable and incorrect use of PPE in the clinical setting.

*A1: In my case, it was handled very differently how the physicians paid attention to it [PPE]. I think that not everyone was equally strict. That was a bit irritating, especially in my first tertial, when you couldn’t really estimate how great the danger was during ward rounds and such. That varied a lot. Some were very strict, while some were not so [strict]. (FG A, 52)*

Similarly, there was inconsistency with respect to the implementation of instructions, which was accompanied by incongruent behaviour on the part of superiors, e.g., junior physicians or senior consultants.

*B4: Then, it was actually a senior physician or so who made a brief comment: “By the way, now you should all wear face shields.“ Then, half of the team wore face shields, and the other half did not. That’s how it went somehow. (FG B, 46)*

Many study participants also mentioned that they felt that medical staff themselves were inexperienced with and unaware of the correct use of PPE.

*A2: But I also had the feeling that some resident physicians were not sure themselves. So, in the COVID-ward, everyone was certain, sure. But in the surgical ward, I heard them saying quite often: “Yes well, I’ve been in the job myself for half a year, I can’t do that [use of PPE] much better than you can. I’ve got to look after myself as well.“ (FG A, 88)*.

In the subcategory of **communication and information flow**, differences among hospitals or wards were also discussed. The study participants emphasized the importance of the proactive communication of the available information regarding PPE, such as new materials or changes in guidelines for use, e.g., during morning rounds in the daily routine in the wards. Overwhelmingly, the flow of information regarding PPE was described as inadequate, especially in normal wards. Another key issue was the level of information and communication regarding patients’ test status. Overall, final-year students did not feel that they were sufficiently involved in the information chain.

*A3: But for me, the really critical point is the overall, individual collegial behaviour of the colleagues in the ward. […] This includes the transmission of important information within the ward, such as, for example, which patients are still waiting for swab results and therefore more risky to transmitting the coronavirus. Such information usually didn’t reach me, and it surely did happen that I walked into such a room without a clue. (FG A, 70)*

With regard to the **time required for PPE instruction and supervision**, it was repeatedly mentioned that overall, sufficient time was frequently not taken for instruction. The study participants attributed this limitation to general stress levels and the high workload of doctors in the wards. The issue of the **influence of the course of the pandemic on the handling of PPE** was also repeatedly mentioned. In this context, COVID-19 was described as a catalyst for practical instruction in the use of PPE.

*B1: Well, without the coronavirus, I don’t think we would have ever learned how to use protective equipment, I suppose. Because you only deal with it to some extent; you learn in your studies a little bit about it, but that’s it. (FG B, 74)*

The influence of vaccination on the use of PPE was also discussed. Most study participants agreed that following the introduction of vaccinations against SARS-CoV-2 for clinical staff, the self-protection aspects of PPE use decreased in importance.

*A5: With the vaccination, one nevertheless felt safer. Then, the [face] mask was sometimes worn more loosely. If you weren’t close to the patient, then you wouldn’t dress up [in PPE] and things like that. That was probably not the case before [the vaccination]. (FG A, 73)*

Following the first breakthrough infections after vaccination, perceived safety was once again described as decreasing, and PPE was again used more thoroughly.

Another aspect of change was introduced by students who had started their final-year placement during the COVID-19 pandemic. They highlighted a tendency to be less willing and motivated to receive explanations or practical introductions related to PPE.

*A4: But I just think that the willingness to explain it [PPE] to them [the final-year students] is not there any longer. Most of them are already vaccinated anyway […] (FG A, 64).*

### Motivation

This category explores the emotional responses of the study participants as well as their drive to use PPE. We identified two subcategories.

Regarding emotional reactions, which are grouped under the motivation category of the COM-B model, the focus was primarily on **fear and uncertainty.** These reactions were triggered by a variety of situations, mainly including exposure to potentially hazardous situations and a lack of materials or clarity pertaining to the use of PPE. Inadequate information flow or a lack of information, e.g., regarding pending test results for patients, were also described as a factor that led to uncertainty.

*A4: […] Then, I was also scared I had been infected […] that was a difficult situation because nobody knew about it. (FG A, 11)*

As an individual driver for the correct use of PPE, the study participants repeatedly focused on the **prioritization of patient safety**. In general, they emphasized the importance of protecting others as a working maxim in the hospital.

*B3: In the hospital, I think, I already went there with the awareness that I was protecting the patients by using protective equipment. Whereas in private, I was more concerned with protecting myself. It’s a different feeling. I think that I did it [using PPE] more consistently in the clinic […] I mean, I am young, I have a good immune system. (FG B, 92)*

**Suggestions for improvement** from the study participants included, in particular, an increase in theoretical and practical teaching regarding PPE during their studies. Participants also expressed a desire for longitudinal linkage, e.g., linking what had already been learned in clinical electives with entry into the final-year placement. With respect to the final-year placement itself, students asked for more hospital- or ward-specific teaching, including a focus on hygiene and PPE.

*A2: Well, I think also, such a proper instruction, one time right on site, in the respective rooms that you are in [is also helpful]. In the Charité learning videos, one may have an airlock, and in the patient rooms on your ward, one may not have something like that at all. […] Then, maybe it could be clearly defined, we do it this way and that way, and then we always do it this way. So, one can always do it this way. Because otherwise, you always do it somehow differently, and that conveys uncertainty. (FG A, 92)*

Another common suggestion was to provide ongoing, low-threshold supervision of PPE use alongside regular feedback.

*B4: But especially if then someone directly offers to show you without you having to ask, because sometimes there’s such an inhibition threshold. Well, I am already in the second tertial now; I can’t just ask at this point anymore how to take the gown off properly.” (FG B, 141)*.

Students would also like to see more support material regarding the use of PPE in the wards, in particular PPE posters illustrating the correct processes for donning and doffing PPE.

*C1: Maybe posters would have helped me. Or something like that, because, as I said, there were a few things that I felt everyone did in the way they thought was right, and then [with a poster], you would know “ah, yes, okay, that is actually correct or not.“ […] so, I’d say that it wouldn’t have been bad as an addition in order to feel somehow on the safe side. (FG C, 73)*

## Discussion

In times of a global pandemic, such as COVID-19, the ready availability and correct use of PPE is an essential and first-line measure to ensure the safety of medical students on clinical placements. In this study, we explored the experiences and perspectives of a group of medical students who used PPE during their final clerkship year during the COVID-19 pandemic and identified several preventable shortcomings in this context. In our approach, we operationalized the correct use of PPE as a trainable behaviour with respect to the enabling factors of capability, opportunities and motivation, based on the COM-B model. In our recommendations for future improvements, we conceptualize responsibility for student safety as a multidimensional construct of shared responsibility among medical schools and their affiliated teaching institutions, teaching and supervising faculty and medical students themselves. Before discussing our findings, we would like to introduce and share the perspective that the implementation of infection prevention and control measures should not be categorized in black and white terms, i.e., as having either been 100% achieved or not achieved. Experience has shown that this field is notoriously imperfect, and improvements in this context benefit from an open, constructive and solution-oriented approach to shortcomings, errors and failures. The following discussion is structured according to the three domains of the COM-B model: capability, opportunity and motivation.

### Role of capability

Regarding the enabling factor capability, participants in our study described shortcomings regarding their PPE learning experiences in terms of both their theoretical and practical components. In particular, shortcomings were identified in preplacement instruction, practical instruction regarding wards and supervision during clinical placements. The study participants found the online instructional video on general infection prevention and control measures, including the correct use of PPE, to be too long and viewed its content as difficult to remember much later in their clinical placements. Another shortcoming identified by study participants was the lack of initial practical instruction in the use of PPE in normal wards. This limitation stood in contrast to their positive evaluations of the practical training they received during placements in COVID-19 intensive care units. This discrepancy is in line with the findings of other studies, such as those from the United Kingdom, where health workers noted that less training took place outside so-called high-risk settings [43]. Before making recommendations for improvement, we would like to note that no best educational practices have been developed for training medical students in the use of PPE. A study from Denmark highlighted that the advantages of an online video-based approach for teaching PPE include the fact that it requires fewer human resources to teach and that it can be viewed repeatedly by students independently at any time [44]. On the other hand, a recent review suggested the superiority of face-to-face training over passive training (e.g., written material or videos) for compliance with the PPE doffing procedure [45]. In addition, the introduction of practical sequences in a blended learning approach has also led to a significant improvement in the PPE doffing skills of paramedic students in Switzerland [46]. We recommend that medical schools and their teaching staff should evaluate and possibly rethink their teaching approaches regarding the correct use of PPE in times of a pandemic, in particular with respect to the role of such equipment in infection prevention and control in general. We also recommend that a co-design approach with medical students should be adopted to facilitate short-term evaluation and elicit suggestions for the improvement and adaptation of the educational materials thus developed.

In the process of learning a new behavioural skill, the study participants also reported gaps in their supervision by supervising physicians regarding the use of PPE to protect themselves and the patients in everyday clinical practice. This experience is consistent with the findings of Barratt et al., who reported that Australian residents face gaps in the appropriate use of PPE and are given little opportunity to develop safe practices in the use of such equipment in the clinical context, which are also due to a lack of supervision [42]. In turn, our study participants notably mentioned that they were more likely to approach nursing staff with questions regarding PPE than their supervising physicians. This approach is not inappropriate, as it can take place in the spirit of interprofessional collaboration. In addition, a report from the United States has documented the fact that nurses more frequently put on and take off PPE than physicians do, as they spend more time engaging in close and in-room patient interactions [47]; therefore, our study participants may have recognized nurses as resources with greater expertise. Regarding supervision, we recommend that medical schools clarify and communicate who is responsible and accountable for supervising the correct use of PPE by medical students throughout the duration of their clinical placements. This group is likely to include both nurses and physicians, and medical schools should agree on a set of rules that should be transparently communicated to all parties involved, to the governance structures of medical schools and to all staff in the wards, including students undergoing placements. In addition, innovative approaches to supervision could be explored to adapt to the changing educational landscape in medical schools with distance and remote learning options.

### Role of opportunity

With regard to the enabling factor opportunity, the study participants reported shortcomings regarding the availability of some PPE components, such as face masks, the varied and inconsistent use of PPE by different healthcare personnel in practice, a lack of time for supervision and a lack of involvement in communication regarding COVID-19 patients. Furthermore, the study participants emphasized the fact that consistency and common guidelines and rules for the use of PPE were important for their learning. The experiences of inadequate availability of PPE and the lack of time for supervision described in this study is consistent with other reports from Europe and the United States [14, 48]. The varied and inconsistent use of PPE by various, often more experienced, healthcare staff is of particular importance, as the students’ learning process with respect to PPE is likely to be shaped and determined by the role models whom they observe and experience in clinical practice. Role models for medical students’ learning include all staff in a ward in general, especially physicians and nurses. In turn, although the study participants experienced the inappropriate use of PPE as a way of disregarding the existing directions, they did not report “speaking up” about this issue themselves. It has been reported in the literature that “speaking-up behaviour” is more commonly related to drug safety than to hygiene [49]. Another study showed that hierarchical structures associated with the fear of negative consequences can strongly influence behaviour and thus put patient safety at risk [50]. These examples and the findings of this study suggest that the issue of “speaking-up behaviour” in the context of ward hierarchies is important both for patient safety and, in particular, for self-protection of medical students. Assuming that medical schools have implemented PPE policies, we recommend the adoption of coherent and multichannel approaches to the implementation of the correct and consistent use of PPE and the use of re-enforcement to raise awareness of the status of each healthcare professional as a role model for students. In addition, the ability of “speaking-up” behaviour should be trained as an integral part of the use of PPE in particular as well as of infection prevention and control measures in general. We therefore recommend that medical students should not only recognize these aspects as part of their learning but also proactively cultivate these skills to promote a shared sense of responsibility for ensuring workplace safety.

### Role of motivation

Regarding the enabling factor motivation, the two ambivalent subthemes that emerged and influenced the student´s use of PPE were uncertainty regarding the safe use of PPE, which was associated with feelings of fear related to contracting COVID-19, and the prioritization of patient safety, which was related to the potential transmission of the virus by the students themselves. The results are consistent with those found by a study conducted in the United Kingdom, where more than half of the participating medical students reported feeling higher levels of uncertainty and anxiety associated with inadequate infection control training and, in particular, inadequate information regarding PPE [24]. Such negative emotional states may have increased the correct and frequent use of PPE but may have hindered their overall learning experience during their clinical placements, an effect which was likely based on the presence of shorter and less intensive patient contact than was the case prior to the pandemic. Regarding the second subtheme, the study participants interestingly prioritized patient safety over the risk of contracting COVID-19 themselves to some extent. This finding is again consistent with the results of studies from the United Kingdom and the United States in which medical students expressed their willingness to accept the risk of infection during the pandemic and to continue working in practice in the safest clinical environment possible [51, 52]. This perspective may be related to the fact that medical students do not generally belong to groups that face the risk of severe disease or death from COVID-19 [53]. It may also pertain to their early professional development in the process of becoming a physician, as this perspective is related to a core ethical attitude on the part of members of the medical profession. Overall, in the area of motivation to use PPE, we found a rich, previously unexplored area for learning, reflection and professional development in clinical placements in general and in times of infectious disease pandemics in particular. We recommend that medical schools, teachers and supervisors, and students actively build on this learning opportunity by emphasizing learning and reflection activities that focus on managing uncertainty and anxiety as well as ethics and professional development when working in potentially hazardous clinical placements. In particular, the role of mental health in medical students should be actively addressed as the COVID-19 pandemic has vividly demonstrated the profound impact such a crisis can exert on their well-being [54].

All in all, the COM-B model provided us with a very valuable framework for analysing in depth the study participants’ experiences with and perspectives on the trainable behaviour “correct use of PPE” in the COVID-19 era as well as the corresponding enabling factors of capability, opportunity and motivation. We were able to identify a number of shortcomings as areas for future improvement and uncovered some interesting new learning opportunities. We also found it to be helpful to combine the COM-B model with the concept of shared responsibility for student safety in clinical placements to encourage the intended behaviour of “correct use of PPE.” In addition, PPE is a good practical example to illustrate the close link between patient safety and occupational safety in clinical placements.

Several methods were used to ensure the quality of the current study: transferability, credibility and member checks [55]. Transferability (external validity) was achieved by providing a detailed description of the sample setting and results to make the study comparable to other settings. The credibility (internal validity) of the current research was ensured through triangulation, a skilful focus group moderation technique and a focus on transparency [56]. To ensure the comprehensiveness of the data, triangulation was achieved by including students from different training sites, i.e., Charité or its teaching hospitals, and different stages of training, i.e., both students in their final clerkship year and students who had already completed their training [56–58]. To enhance reflexivity, the principle researcher JS kept a diary during the study period. In this case, the researcher can be seen as an outsider who did not teach or examine the student participants either before or after the focus groups were conducted. Validity was also ensured through the constructive alignment among the research question of how medical students experienced training regarding and the use of PPE during the COVID-19 pandemic, the epistemology of social constructivism, according to which participants are viewed as actively and jointly constructing experiences and meanings, and the focus group method, which facilitates interaction and generates rich data [31, 59].

This work has limitations. The study focuses on a single centre, and its generalizability to other contexts is unknown. It reports on the experiences and perspectives of participating medical students and may have limited generalizability to the student cohort as a whole. Participation in the study was voluntary, and the student recruitment process may have introduced selection bias, such as a potentially favouring recruitment of students who were very satisfied or dissatisfied with their clerkship or practical training. Another limitation may arise be the group setting of the discussions sessions that may have restricted what experiences the participants were willing to share. We invited only medical students to participate, and other groups, such as teachers, supervisors, nurses or curriculum managers, should be approached in future studies.

## Conclusions

The COVID-19 pandemic is still ongoing and will not be the last pandemic to affect student safety and learning during clinical placements. The COM-B model allows us to identify shortcomings regarding the “correct use of PPE” systematically. Alongside the concept of shared responsibility for student safety, we have been able to derive recommendations for improvement relating both to the enabling factors of capability, opportunity and motivation and to the allocation of specific responsibilities to the medical school as an organization, to teachers and supervisors, or to medical students themselves. Our approach and findings may guide and stimulate other medical schools and faculties to explore and analyse the components of student safety in clinical settings in general and in times of infectious pandemics in particular.

### Electronic supplementary material

Below is the link to the electronic supplementary material.

Supplementary Material 1

## Data Availability

The dataset used and analysed during this study is not publicly available due to ethical and regulatory restrictions but is available from the corresponding author upon reasonable request.

## Abbreviations

PPE: Personal Protective Equipment
COM-B: Capability, Opportunity, Motivation – Behaviour
