# Supplementary material for: Personal protective equipment and medical students in times of COVID-19: experiences and perspectives from the final clerkship year
Source: BMC Med Educ. 2023 Oct 26;23:806. doi: 10.1186/s12909-023-04784-2 (PMC10605960; doi:10.1186/s12909-023-04784-2)
Supplement: Supplementary file 1 — Supplementary Material 1 [file 12909_2023_4784_MOESM1_ESM.docx]

**Additional file: Discussion guide for focus groups**

**Subject:** "How do medical students experience training regarding personal protective equipment (PPE) and the use of such equipment during their final clerkship year?"

| **Content** | **Time** |
| --- | --- |
| *Before:*   - Starting the meeting on MS Teams - Possibility of a technical check-in for participants | |
| **Opening & welcome** | 15 min |
| - Welcome to all participants - Introduction of the moderator and a brief round of introductions of all participants - Brief overview of the time schedule (total duration: approx. 1.5 h), agreement on common "rules of conversation" (minimize background noise, etc.) - Note on audio recording and data protection/ethics vote *(start recording)*   **Introduction to the methodology and topic of the "focus group discussion"**   - This discussion will focus on experiences with personal protective equipment as well as teaching regarding and the use of such equipment. These group discussions are part of a qualitative study (for a Master’s thesis) exploring the experiences of final-year medical students regarding occupational safety, especially with respect to personal protective equipment training during the final clerkship year in the context of the COVID-19 pandemic. Accordingly, all experiences pertaining to this topic from final-year medical students are part of the focus of these discussions. - I will read short introductory questions on three overall topics to initiate the discussion. The aim of this so-called focus group discussion is also for you to complement each other and engage in conversation with each other. As moderator, I will then ask further questions if necessary. - Is the abbreviation for personal protective equipment (PPE) known? - Are there any questions about the topic or the procedure? If not, then we shall start. | |
| 1. **Topic: Experiences with teaching regarding PPE** | 25 min |
| - Entry question:   - What is your experience with instructions regarding personal protective equipment (PPE) in general? - Possible follow-up questions:   - Can you describe that in more detail?   - In what form was there "verification" of what was learned? (In the form of signatures? Certificates?)   - What were the biggest challenges with instructions (theoretical/practical) regarding PPE?   - Do you have the impression that something has changed with regard to PPE training for fellow students who are now starting their final year? What changes do you recognize?   - What differences between the "pre-Corona time" and the "Corona time" to teaching with PPE have you experienced?   - What differences did you encounter in the instructions regarding PPE for other occupational groups? Especially with regard to nursing (nursing students)? | |
| 1. **Topic: Experiences with the use of PPE during the final-year clerkship** | 20 min |
| - Entry question:   - How did you experience the use of personal protective equipment (PPE) during your final clerkship year? - Possible follow-up questions:   - What were some particular key experiences with using PPE during your final-year clerkship?   - How did supervision of final-year students take place? Was the safe use of PPE proactively addressed by supervising physicians?   - What form of supporting material was provided (posters, video tutorials)? Which did you use? Which did you find helpful?   - Who would you contact with questions regarding the adequate use of PPE?   - What observations of colleagues (physicians, nurses) regarding the use of PPE have stuck in your mind?   - What changes regarding the use of PPE have you observed since filtering facepiece (FFP-2) masks have been recommended and used in the general population? | |
| 1. **Topic: Suggestions for improvement** | 20 min |
| - In retrospect, what form of PPE instruction do you consider appropriate?   - What would you have liked to see? When should induction take place, and how can adequate use be ensured? As an objective structured clinical examination (OSCE) for advancement to the final clerkship year? - What ideas do you have regarding how to strengthen final-year students’ safety further? (Focus on PPE) - As a resident, how will you instruct final-year students in your unit in the use of PPE? | |
| **Conclusion & time buffer** | 10 min |
| - Closing question:   - If you could change one thing regarding instruction on PPE, what would you change? - Thanks for participation & farewell | |
